# Supplementary material for: Experiences of Female Childhood Cancer Patients and Survivors Regarding Information and Counselling on Gonadotoxicity Risk and Fertility Preservation at Diagnosis: A Systematic Review
Source: Cancers (Basel). 2023 Mar 23;15(7):1946. doi: 10.3390/cancers15071946 (PMC10093478; doi:10.3390/cancers15071946)
Supplement: Supplementary file 1 [file cancers-15-01946-s001.zip › cancers-2242005-supplementary.pdf]

Supplementary Materials:

Supplementary Tables:

Table S1. (A). Search terms PubMed Database. **(B).** Search syntax PubMed Database
Table S2. (A). Search terms Embase Database. **(B).** Search syntax Embase Database
Table S3. Evidence Table Crawshaw, Glaser, Hale, & Sloper. 2009.(1)
Table S4. Evidence Table Jardim, Lopes-Junior, Nascimento, Neves, & de Lima. 2021.(2)
Table S5. Evidence Table Kim, & Mersereau. 2015.(3)
Table S6. Evidence Table Oosterhuis, Goodwin, Kiernan, Hudson, & Dahl. 2008.(4)
Table S7. Evidence Table Sandheinrich, Wondmeneh, Mohrmann, Gettinger, Henry, & Hayashi. 2018.(5)
Table S8. Evidence Table Wright, Coad, Morgan, Stark, & Cable. 2014.(6)
Table S9. Evidence Table Zarnegar, Goseingfiao, Rademaker, Casey, & Albritton. 2018.(7)

Table S1. (A). Search terms PubMed Database. **(B).** Search syntax PubMed Database.

**(A)**		
**Research question**	**Keyword**	**Search terms**
Population	Cancer	neoplasm[MeSH] OR cancer[MeSH] OR tumor[MeSH] OR tumour[MeSH] OR neoplasia[MeSH] OR malignancy[MeSH] OR cancer*[tiab] OR tumor*[tiab] OR tumour*[tiab] OR neoplas*[tiab] OR malignan*[tiab] OR oncolog*[tiab]
	Childhood/ children (age 0-19 years)	child[MeSH] OR infant[MeSH] OR pediatric[MeSH] OR paediatric[MeSH] OR child*[tiab] OR infant*[tiab] infanc*[tiab] OR pediatric*[tiab] OR paediatric*[tiab] OR adolescent[MeSH] OR teenage[tiab] OR adolescen*[tiab]
	Gonadal damage, gonadotoxicity, infertility	((gonad*[tiab] OR ovary[tiab] OR ovari*[tiab]) AND (damage*[tiab] OR fail*[tiab] OR insufficien*[tiab] OR function*[tiab] OR toxic*[tiab])) OR (fertility[MeSH] OR fecundity[MeSH] OR fertil*[tiab] OR onco-fertil*[tiab] OR oncofertil*[tiab] OR fecund*[tiab] OR reproduc*[tiab] OR infertility[MeSH] OR subfertility[MeSH] OR sub-fertility[MeSH] OR infertil*[tiab] OR subfertil*[tiab] OR sub-fertil*[tiab])
Context	Counselling	counselling[MeSH] OR counsel*[tiab] OR specialist[MeSH] OR gynaecol*[tiab] OR gynecol*[tiab] OR specialist*[tiab] OR fertility preservation[MeSH] OR preservation, fertility[MeSH] OR preser*[tiab] OR educat*[tiab] OR inform*[tiab] OR patient education[MeSH]
Outcome	Experience	experience*[tiab]
	Concern	concern*[tiab]
	Regret	regret[MeSH] OR regrets[MeSH] OR regret*[tiab]
	Satisfaction	satisfaction[MeSH] OR personal satisfaction[MeSH] OR patient satisfaction[MeSH] OR satisf*[tiab]
	Decision-making	decision making[MeSH] OR thinking[MeSH] OR choice[MeSH] OR decision*[tiab] OR think*[tiab] OR choice*[tiab]

**(B) Search syntax**
((((neoplasm[MeSH] OR cancer[MeSH] OR tumor[MeSH] OR tumour[MeSH] OR neoplasia[MeSH] OR malignancy[MeSH] OR cancer*[tiab] OR tumor*[tiab] OR tumour*[tiab] OR neoplas*[tiab] OR malignan*[tiab] OR oncolog*[tiab]) AND (child[MeSH] OR infant[MeSH] OR pediatric[MeSH] OR paediatric[MeSH] OR child*[tiab] OR infant*[tiab] infanc*[tiab] OR pediatric*[tiab] OR paediatric*[tiab] OR adolescent[MeSH] OR teenage[tiab] OR adolescen*[tiab])) AND (((gonad*[tiab] OR ovary[tiab] OR ovari*[tiab]) AND (damage*[tiab] OR fail*[tiab] OR insufficien*[tiab] OR function*[tiab] OR toxic*[tiab])) OR (fertility[MeSH] OR fecundity[MeSH] OR fertil*[tiab] OR onco-fertil*[tiab] OR oncofertil*[tiab] OR fecund*[tiab] OR reproduc*[tiab] OR infertility[MeSH] OR subfertility[MeSH] OR sub-fertility[MeSH] OR infertil*[tiab] OR subfertil*[tiab] OR sub-fertil*[tiab]))) AND (counselling[MeSH] OR counsel*[tiab] OR specialist[MeSH] OR gynaecol*[tiab] OR gynecol*[tiab] OR specialist*[tiab] OR fertility preservation[MeSH] OR preservation, fertility[MeSH] OR preser*[tiab] OR educat*[tiab] OR inform*[tiab] OR patient education[MeSH])) AND (((((concern*[tiab]) OR (decision making[MeSH] OR thinking[MeSH] OR choice[MeSH] OR decision*[tiab] OR think*[tiab] OR choice*[tiab])) OR (experience*[tiab])) OR (regret[MeSH] OR regrets[MeSH] OR regret*[tiab])) OR (satisfaction[MeSH] OR personal satisfaction[MeSH] OR patient satisfaction[MeSH] OR satisf*[tiab]))

Table S2. (A). Search terms Embase Database. **(B).** Search syntax Embase Database.

**(A)**		
**Research question**	**Keyword**	**Search terms**
Population	Cancer	'malignant neoplasm'/exp OR 'childhood cancer'/exp OR neoplas*:ti,ab,kw OR cancer*:ti,ab,kw OR tumor*:ti,ab,kw OR tumour*:ti,ab,kw OR malignan*:ti,ab,kw OR oncolog*:ti,ab,kw
	Childhood/ children (age 0-19 years)	child/exp OR infant/exp OR pediatric/exp OR adolescent/exp OR 'childhood cancer survivor'/exp OR child*:ti,ab,kw OR infant*:ti,ab,kw OR infanc*:ti,ab,kw OR pediatric*:ti,ab,kw OR paediatric*:ti,ab,kw OR adolescen*:ti,ab,kw OR teenage*:ti,ab,kw
	Gonadal damage, gonadotoxicity, infertility	'gonadal damage'*:ti,ab,kw OR ovary*:ti,ab,kw OR toxicity*:ti,ab,kw OR insufficiency*:ti,ab,kw OR 'ovarian insufficiency'*:ti,ab,kw OR fertility/exp OR infertility/exp OR subfertility/exp OR fertil*:ti,ab,kw OR onco-fertil*:ti,ab,kw OR oncofertil*:ti,ab,kw OR fecundity*:ti,ab,kw OR reproduc*:ti,ab,kw OR infertil*:ti,ab,kw OR subfertil*:ti,ab,kw OR sub-fertil*:ti,ab,kw
Context	Counselling	counseling/exp OR 'medical specialist'/exp OR gynecologist/exp OR 'fertility specialist'/exp OR 'fertility preservation'/exp OR 'patient education'/exp OR counsel*:ti,ab,kw OR gynecol*:ti,ab,kw OR specialist*:ti,ab,kw OR preser*:ti,ab,kw OR education*:ti,ab,kw OR inform*:ti,ab,kw
Outcome	Experience	experience*:ti,ab,kw
	Concern	concern*:ti,ab,kw
	Regret	regret/exp OR regret*:ti,ab,kw
	Satisfaction	satisfaction/exp OR 'patient satisfaction'/exp OR satisf*:ti,ab,kw
	Decision-making	'decision making':ti,ab,kw OR think*:ti,ab,kw OR 'patient satisfaction':ti,ab,kw OR 'decision making'/exp OR decision*:ti,ab,kw OR choice*:ti,ab,kw

**Search syntax**
('malignant neoplasm'/exp OR 'childhood cancer'/exp OR neoplas*:ti,ab,kw OR cancer*:ti,ab,kw OR tumor*:ti,ab,kw OR tumour*:ti,ab,kw OR malignan*:ti,ab,kw OR oncolog*:ti,ab,kw) AND (child/exp OR infant/exp OR pediatric/exp OR adolescent/exp OR 'childhood cancer survivor'/exp OR child*:ti,ab,kw OR infant*:ti,ab,kw OR infanc*:ti,ab,kw OR pediatric*:ti,ab,kw OR paediatric*:ti,ab,kw OR adolescen*:ti,ab,kw OR teenage*:ti,ab,kw) AND ('gonadal damage'*:ti,ab,kw OR ovary*:ti,ab,kw OR toxicity*:ti,ab,kw OR insufficiency*:ti,ab,kw OR 'ovarian insufficiency'*:ti,ab,kw OR fertility/exp OR infertility/exp OR subfertility/exp OR fertil*:ti,ab,kw OR onco-fertil*:ti,ab,kw OR oncofertil*:ti,ab,kw OR fecundity*:ti,ab,kw OR reproduc*:ti,ab,kw OR infertil*:ti,ab,kw OR subfertil*:ti,ab,kw OR sub-fertil*:ti,ab,kw) AND (counseling/exp OR 'medical specialist'/exp OR gynecologist/exp OR 'fertility specialist'/exp OR 'fertility preservation'/exp OR 'patient education'/exp OR counsel*:ti,ab,kw OR gynecol*:ti,ab,kw OR specialist*:ti,ab,kw OR preser*:ti,ab,kw OR education*:ti,ab,kw OR inform*:ti,ab,kw) AND ('decision making':ti,ab,kw OR think*:ti,ab,kw OR 'patient satisfaction':ti,ab,kw OR 'decision making'/exp OR regret/exp OR satisfaction/exp OR 'patient satisfaction'/exp OR concern*:ti,ab,kw OR decision*:ti,ab,kw OR choice*:ti,ab,kw OR experience*:ti,ab,kw OR regret*:ti,ab,kw OR satisf*:ti,ab,kw)

Table S3: Evidence table Crawshaw, Glaser, Hale, & Sloper. 2009.(1)

What are the experiences of female patients regarding fertility counselling during childhood cancer treatment?	
Crawshaw MA, Glaser AW, Hale JP, Sloper P. Male and female experiences of having fertility matters raised alongside a cancer diagnosis during the teenage and young adult years. Eur J Cancer Care (Engl). 2009;18(4):381-90.	
**Study**	**Participants**
<u>Study type:</u><br>Qualitative study by grounded theory.<br><br><u>Study era:</u><br>2004-2009.<br><br><u>Country:</u><br>United Kingdom.<br><br><u>Data collection:</u><br>In-depth single interviews, at a time, place, and length and with supporters of participants' choosing. Tape-recorded and transcribed.<br><br><u>Data analysis:</u><br>Preliminary coding frame. Multiple transcribers. AtlasTi. Codes added to preliminary frame. Patterns emerged after discussions. Data presented in narrative form.	<u>Non-participants:</u><br>NR.<br><br><u>Participants:</u><br>Response rate 35%. *N* = 38. Male and female adolescent childhood cancer survivors, recruited from three regional paediatric oncology units in the North of England.<br>Female childhood cancer survivors = 17/38 = 45%. Female childhood cancer survivors <19 years = 13/17.<br>Types of cancer (total, not female specific*):<br>• 14 Sarcoma 37%;<br>• 9 Lymphoma 24/16%;<br>• 6 Leukaemia 16%;<br>• 5 Germ cell tumours 13%;<br>• 4 Central nervous system tumours 11%.<br>HR patients: NR. *including information from Crawshaw *et al.* 2010.<br><br><u>Age at study (range):</u><br>Female <19 years: median 20 years (16-26).<br>Including female >18: median 21 years (16-26).<br>All male and female: median 21 years (16-30). At least 1 year after diagnosis.<br><br><u>Age at diagnosis (range):</u><br>Female <19 years: median 15 years (11-18).<br>Including female >18 (3 19 and 1 20 years at diagnosis): median 15 years (11-20).<br>All male and female: 15 years (11-20).<br><br><u>Informed/counselled by:</u><br>Consultant paediatric/adolescent oncologists, nurses and social workers. Fertility matters raised at diagnosis, some (*n* = NR) did not remember whether this was before or after start treatment.
**General remarks**	**General strengths and limitations**
Aim: male and female adolescent cancer patients' experiences of having fertility and associated decision-making matters raised at diagnosis.<br>Teenagers classified as <21 years.<br>Inclusion criteria included being aware their fertility might have been affected and that they were diagnosed between 13 and 20 years old, not receiving treatment at the time of being approached about participation in this research.<br>Grounded theory: systematic methodology construction of hypotheses and theories through the collecting and analysis of data, inductive reasoning.<br>Paediatric/adolescent oncologist informed about fertility at/around diagnosis (nurse/social worker involvement was limited).<br>No participant counselled by gynaecologists. Assisted conception centre staff involved for fertility preservation.<br>Little prior knowledge of fertility impairment.<br>39% residual impairment or undergoing further investigations/treatments.	<u>Strengths:</u><br>• Interviews at a time, place and length of participants' choosing with supporters if desired;<br>• In-depth data;<br>• Multiple transcribers;<br>• Iterative data analysis.<br><u>Limitations:</u><br>• Small sample size, low representativeness and reliability;<br>• Results (e.g. concerning regrets after fertility preservation offer) not described very clearly, thus difficult to draw conclusions from this;<br>• Only patients included that recalled fertility discussion;<br>• Many results not female specific or vaguely described, often no frequencies given, thus difficult to interpret;<br>• Same patients as Crawshaw *et al.* 2010.

Bias risk	
**I. An explicit account of theoretical framework and/or the inclusion of a literature review which outlined a rationale for the intervention.**<br>Yes: Grounded theory used as a theoretical framework to gather information of the experiences of participants.<br><br>**II. Clearly stated aims and objectives.**<br>Yes: 'This study aims to provide the first report of the male and female adolescent cancer patients' experiences of having fertility and associated decision-making matters raised at diagnosis.'<br><br>**III. A clear description of context which includes detail on factors important for interpreting results.**<br>Yes: Information was reported on where patients were recruited and data was collected, when data was collection. Data collection and analysis was described clearly. Ethical approval was obtained from the National Health Service ethics and research governance. However, it was not reported who collected data.<br><br>**IV. A clear description of sample.**<br>Yes: Age at diagnosis, study, race, sexuality, cancer types, living situation, education.	**V. A clear description of methodology, including systematic data collection methods.**<br>No: Data analysis described, but methodology very limited, no information on outcome measures, data collection and qualitative interviews.<br><br>**VI. Analysis of the data by more than one researcher.**<br>Yes: It is not reported that researchers analyzed independently, but they 'had several meetings on findings'.<br><br>**VII. The inclusion of sufficient original data to mediate between data and interpretation.**<br>Yes: No tables provided, but discussion is based on given results and citations of texts from respondents were included.<br><br>Six of seven criteria met: <u>low</u> risk of bias.

Main outcomes
<u>Outcome definitions:</u><br>Satisfaction not defined but 'support for' interpreted as 'satisfied with'.<br>Regrets not defined but 'times of later preoccupation with the circumstances of their decision', 'questioned the wisdom of the offer being made' and 'more difficult to cope with' interpreted as 'regrets'.<br>Concerns NR.<br><u>Results:</u><br>Experiences:<br>Most of the women were informed at diagnosis (*n* = NR). Not female specific: Professional sensitivity, friendliness, lack of embarrassment, clear information and a choice who is present during conversation led to a positive experience. CCS preferred 1 or 2 professionals and a small number of family/parental supporters. CCS welcomed hearing that they make the final decision after advice and encouragement from professionals. Some (*n* = NR) appreciated the conversation about fertility preservation as professional believe that they have a future. However, several (*n* = NR) did not like that after a life-threatening diagnosis they needed to make choices about future parenthood. None remembered being offered counselling. Females CCS (*n* = NR) wanted to receive information independent on the risk of gonadal damage or options for fertility preservation. Women require complex information, especially when preservation is offered, comprehension less successfully achieved (vaguely described). "A few who remembered being told by a doctor that their periods might be affected, at least temporarily, had not understood until much later that this meant that their fertility may be affected – that is, they had not associated their menstrual cycle with their reproductive system". Some (*n* = NR) female CCS prefer an explanation why oocyte cryopreservation is not an option to prevent the negative experience of finding out later that this option exists.<br>Satisfaction:<br>Great support for information at diagnosis (general). Many (*n* = NR, general) valued parental support to help remember and interpret information. Professionals talk directly to patients leads to satisfaction (female, 15yo at diagnosis). Females CCS (*n* = NR) would be unsatisfied and lose trust in the health care professionals if the information would be withheld, delayed or later told in an unplanned way. Being told ensured they felt aware of the challenges ahead and could develop coping mechanisms. The 5 who were offered fertility preservation found timing of fertility preservation difficult, but understandable. Offer of fertility preservation welcomed. Most (*n* = NR, general) found broad information satisfied, providing age-appropriate, room for questions and more detailed discussions later. Inadequate, confusing and/or poorly delivered information impacts negatively, including reproductive understanding. The information given to females was often unsatisfactory (*n* = NR): information given was sometimes wrong, the information was not always understood, not enough explanation is given about why FP was no option, relation between menstrual cycle and fertility was often not clearly explained.<br>Regrets:<br>5 were offered fertility preservation, 1 had ovaries 'tied up', experience and fertility status at time of the study: NR. 3 declined oocyte cryopreservation to avoid delaying treatment, of which 2 were later infertile and 1 unclear status, which "led to times of later preoccupation with the circumstances of their decision". 1 was offered protection from radiotherapy, but declined because the risk was low at the time. "She later questioned the wisdom of the offer being made and felt that this made her later (unpredicted) infertility more difficult to cope with". Doubts of timing of offer.<br>Concerns: NR.

Table S4: Evidence table Jardim, Lopes-Junior, Nascimento, Neves, & de Lima. 2021.(2)

What are the experiences of female patients regarding fertility counselling during childhood cancer treatment?	
Jardim FA, Lopes-Junior LC, Nascimento LC, Neves ET, de Lima RAG. Fertility-Related Concerns and Uncertainties in Adolescent and Young Adult Childhood Cancer Survivors. J Adolesc Young Adult Oncol. 2021;10(1):85-91.	
**Study**	**Participants**
<u>Study type:</u><br>Qualitative study.<br><br><u>Study era:</u><br>2016.<br><br><u>Country:</u><br>Brazil.<br><br><u>Data collection:</u><br>Semi-structured interview, predesigned, tested script. Developed and reviewed by research team (clinical nurses, oncology specialists). Extensive review of relevant literature on fertility-relate issues in AYA cancer survivors.<br><br><u>Data-analysis:</u><br>Interviews, by one author, recorded and transcribed. Multiple transcribers. Data analysed using thematic analysis:<br>1. Approaching transcripts;<br>2. Identifying, coding;<br>3. Revising codes, constructing themes;<br>4. Revising, refining themes;<br>5. Naming thematic axes;<br>6. Interpreting themes.<br>Consensus discrepancies. Certified, validated themes.	<u>Non-participants:</u><br>*N* = 2. 1 having due to feelings of embarrassment, topic was difficult assimilate and uncomfortable with sharing fertility information with partner. 1 mother declined fertility is delicate subject difficult to assimilate for her daughter. No other data reported on non-participants.<br><br><u>Participants:</u><br>*N* = 24. Male and female childhood cancer survivors from oncohematology outpatient clinic, university hospital in Brazil.<br>Female childhood cancer survivors = 11/24 = 46%, but contains female specific data.<br>HR patients: NR.<br>Type of cancers (not female specific):<br>• 7 Solid tumours (osteosarcoma, Ewing's sarcoma) 29.17%;<br>• 3 Lymphoma 12.5%;<br>• 8 Leukaemia 33.33%;<br>• 5 Brain tumour 20.83%;<br>• 1 Adrenal tumour 4.17%.<br><br><u>Age at study (range) (not female specific):</u><br>Mean 20.38 years, SD 1.76 (18-24).<br><br><u>Age at diagnosis (range) (not female specific):</u><br>Mean 8.13 years, SD 3.75 (1-15).<br><br><u>Informed/counselled by:</u> NR.<br>Some CCS (*n* = 4) reported their mothers were informed by healthcare professionals, the doctor (not further specified). Timing NR.
**General remarks**	**General strengths and limitations**
Aim: uncover the fertility-related concerns and uncertainties in adolescent and young adult (AYA) childhood cancer survivors.<br>Definition: Paediatric cancer survivor = survived for >5 years from the last evidence of disease and discontinued therapy for ~2 years.<br>Inclusion criteria: (1) childhood cancer survivor (18–24 years); (2) having completed cancer treatment for >5 years, disease-free; (3) diagnosed between 0 and 18 years; and (4) received chemotherapy or radiotherapy treatment.<br>Time since diagnosis (not female specific): Mean 10.42 years, SD: 3.16; range 5-15 years.<br>Information fertility status information difficult from medical records. Information indirectly identified from medical records when the referral to the gynaecologist was checked.	<u>Strengths:</u><br>• Interview script developed and reviewed by research team (clinical nurses and oncology specialists), after extensive review of relevant literature on fertility-related issues in AYA cancer survivors.<br>• Female specific data;<br>• Findings useful in understanding fertility-related concerns and uncertainty of AYA childhood cancer survivors in developing countries;<br>• Findings offer novel insights;<br><u>Limitations:</u><br>• Participants recruited from one specialized outpatients ward in oncology-haematology department at university hospital in Brazil;<br>• Lack of reliability of participants' ability to recall information with regard to previous experiences with cancer at young age.

<table>
<tr><th colspan="2">Bias risk</th></tr>
<tr><td>
I. An explicit account of theoretical framework and/or the inclusion of a literature review which outlined a rationale for the intervention.
Yes: An interview schedule was developed by an experienced multidisciplinary team following an extensive review of the relevant literature on fertility-related issues in AYA cancer survivors.

II. Clearly stated aims and objectives.
Yes: ‘Hence, this study aimed to uncover the fertility-related concerns and uncertainties of AYA childhood cancer survivors.’

III. A clear description of context which includes detail on factors important for interpreting results.
Yes: Information is reported on who collected data, where patients was recruited, when data was collected and how data was collected. Ethical approval was obtained from the Institutional Review Board of the University of São Paulo. No information is reported on how data was collected.

IV. A clear description of sample.
Yes: Age at diagnosis and study, education, types of cancer.
</td><td>
V. A clear description of methodology, including systematic data collection methods.
Yes: Information is reported on data collection, including development of an interview script, and data analysis, including reporting of findings. No information is reported on outcome measures.

VI. Analysis of the data by more than one researcher.
Yes: Two investigators coded data from interviews independently.

VII. The inclusion of sufficient original data to mediate between data and interpretation.
Yes: Tables provided with findings of responses from participants, including quotations. Discussion based on results.

Seven of seven criteria met: <u>low</u> risk of bias.
</td></tr>
<tr><th colspan="2">Main outcomes</th></tr>
<tr><td colspan="2">
<u>Outcome definitions:</u>
Satisfaction not defined, but ‘would receive information about the possible effects of cancer treatment on fertility’ interpreted as ‘satisfaction’.
Regrets NR.
Concerns not defined, but ‘feelings of sadness in the inability to become mothers’, ‘uncertainty of the potential adverse effects of treatment on fertility’ and ‘sharing reproductive incapacity associated with fear’ interpreted as concerns.
4 themes: (1) knowledge about fertility, (2) emotional impact and fertility-related uncertainty, (3) sharing the possible risk of infertility with partners, and (4) need for information on possible loss of fertility.
<u>Results:</u>
Experience:
4 women reported mother was informed, 2 because they were very young, age at study: 18 and 20yo. 4 women reported doubts and misconceptions about (in)fertility. 5 women are sad because of inability to become a mother, 7 women are uncertain about reproductive status. 5 women hope to have biological children, 10 women need more information. 1 female experienced miscommunication.
Satisfaction:
In general 75% says more information is needed, in women this is 10/11. Majority wants to receive fertility information.
Regrets: NR.
Concerns:
Feelings of sadness with possible childlessness (n = 5). Coexist with concerns regarding potential infertility. 11 women have no difficulty telling partner about possible infertility. However, concerns are associated with fear of abandonment.
</td></tr>
</table>

Table S5: Evidence table Kim, & Mersereau. 2015.(3)

<table>
<tr><th colspan="2">What are the experiences of female patients regarding fertility counselling during childhood cancer treatment?</th></tr>
<tr><td colspan="2">Kim J, Mersereau JE. A pilot study about female adolescent/young childhood cancer survivors' knowledge about reproductive health and their views about consultation with a fertility specialist. Palliat Support Care. 2015;13(5):1251-60.</td></tr>
<tr><th>Study</th><th>Participants</th></tr>
<tr><td>Study type:
Pilot study, cross-sectional, quantitative study.

Study era:
2014-2015.

Country:
United States of America.

Data collection:
Web-based survey. Questionnaire developed by fertility specialists, reviewed by physicians, survey experts and non-physician staff.

Data-analysis:
Descriptive statistics. Univariate analysis for prognostic variables for positive views reproductive counselling. T tests, Fisher's exact test. SAS statistical software.</td><td>Non-participants:
N = 60. No differences between participants and non-participants regarding current age, age at diagnosis and type of cancer. 57 eligible no correct address available.

Participants:
N = 56. Response rate: 48%. Female childhood and adolescent cancer survivors, who completed cancer treatment with currently no evidence of disease.
HR patients: NR.
Types of cancer:
• Leukaemia 34%;
• Lymphoma 16%;
• Brain tumour 5%;
• Kidney cancer 4%;
• Bone cancer 5%;
• Soft tissue cancer 5%;
• Others 36%.

Age at study (range):
Mean 26 years (SD: 4) (22-30).

Age at diagnosis (range):
Mean 13 years (SD: 6) (7-19).

Informed/counselled by (patients and/or parents/guardians; this is not specified):
At diagnosis: 31/56 discussion fertility with healthcare professionals, 5/56 discussion with fertility specialist.
After treatment: 31/56 discussion fertility with healthcare professionals. , 3/56 discussion with fertility specialist.</td></tr>
<tr><th>General remarks</th><th>General strengths and limitations</th></tr>
<tr><td>Aims: measure female CCSs' knowledge about their reproductive health and their exposure to and views about reproductive counselling (RC). Mean knowledge score of participants was 32%. Mean knowledge score in women pursued reproductive counselling (*n* = 5) not different from in women who did not pursue reproductive counselling (p = 0.25).
Inclusion criteria: female CCSs (18 - 45 years) diagnosed with cancer <21 years between (1994 and 2010) and currently no evidence of disease.
5 women (9%): premature menopause, mean age at menopause 22 (SD: 3) years.
16 women (29%): irregular periods.</td><td>Strengths:
• Results of pilot study preliminary data to discuss fertility information with patients (females only) at diagnosis or after treatment;
• Questionnaire developed by fertility specialists, reviewed by physicians and survey experts for question content and non-physician staff for clarity.
Limitations:
• Small number of participants from single institution, low response rate;
• Survey based, no interviews;
• Results not split by those who were informed by professional or their parents.</td></tr>
</table>

<table>
<tr><th colspan="2">Bias risk</th></tr>
<tr><td>
I. An explicit account of theoretical framework and/or the inclusion of a literature review which outlined a rationale for the intervention.
No: The questionnaire was developed by fertility specialists familiar with fertility preservation and the questions were reviewed, but it is not mentioned that a theoretical framework of literature review was used for this.

II. Clearly stated aims and objectives.
Yes: ‘This paper reports the results of a single-center pilot study that aimed to measure female CCSs’ knowledge about their reproductive health as well as their exposure to and views about reproductive counselling.’

III. A clear description of context which includes detail on factors important for interpreting results.
Yes: Information was reported on how participants were recruited, when data was collected and how data was collected. Ethical approval was granted by the UNC institutional review board. No information was reported on who collected data.

IV. A clear description of sample.
Yes: Age at diagnosis and study, BMI, race, education, types of cancer and cancer treatment and reproductive history.
</td><td>
V. A clear description of methodology, including systematic data collection methods.
Yes: Information is reported on data collection, including development of the questionnaire. Outcome measures are described. Data and statistical analysis is reported.

VI. Analysis of the data by more than one researcher.
Not applicable to this study.

VII. The inclusion of sufficient original data to mediate between data and interpretation.
Yes: Tables provided with findings. Quotations are not included. Discussion based on results.

Five of seven criteria met: <u>low</u> risk of bias.
</td></tr>
<tr><th colspan="2">Main outcomes</th></tr>
<tr><td colspan="2">
<u>Outcome definitions:</u>
Satisfaction defined as ‘their views about the content and quality of information that was provided’ and ‘A positive view about reproductive counselling with a fertility specialist was defined as having pursued RC or as being willing to have pursued RC if an opportunity had arisen. A negative view of RC was defined as not being interested in pursuing RC.’
Regrets NR.
Concerns defined as ‘decreased fertility next most important cancer-related concern’.
<u>Results:</u>
Experience:
44% (n = 25) of CCS did not recall that they or parent/guardian discussed reproductive issues and options with healthcare providers (the oncology team and/or a fertility specialist) at the time of cancer diagnosis or after treatment. Suggested topics to be discussed during counselling after treatment: loss of fertility, future childbearing plans, and impact of cancer/cancer treatment on future pregnancy outcome.
Satisfaction:
31/56 informed at diagnosis by healthcare professional, 5/31 received FC. 4/5 women counselled at diagnosis satisfied. 21/51 welcomed counselling at diagnosis. The most common reason (n = NR) why RC would not have been helpful was: too young to discuss reproductive issues. 16/56 satisfied with received fertility information at diagnosis.
31/56 had at least 1 discussion after treatment (not RC), 48% once a year, 23% less than once a year, and 16% once. 3 counselled with RC after treatment: 2 satisfied. 29/56 would pursue reproductive counselling. 22/56 satisfied with received fertility information after treatment. 40/56 had a positive view about reproductive counselling and thus had or would have pursued RC had the opportunity arisen. Majority not satisfied with information: diagnosis; 40/56 (71%) not satisfied (16 satisfied) and end of treatment; 34/56 (60%) not satisfied (22 satisfied).
Regrets: NR.
Concerns:
Decreased fertility next most important cancer-related concern after survival and risk of cancer recurrence.
</td></tr>
</table>

Table S6: Evidence table Oosterhuis, Goodwin, Kiernan, Hudson, & Dahl. 2008.(4)

What are the experiences of female patients regarding fertility counselling during childhood cancer treatment?	
Oosterhuis BE, Goodwin T, Kiernan M, Hudson MM, Dahl GV. Concerns about infertility risks among pediatric oncology patients and their parents. Pediatr Blood Cancer. 2008;50(1):85-9.	
**Study**	**Participants**
Study type:<br>Quantitative study, survey.<br><br>Study era:<br>2005-2008.<br><br>Country:<br>United States of America.<br><br>Data collection:<br>Survey, evaluating concerns about fertility-related side effects of cancer treatment and satisfaction provided information.<br><br>Data-analysis:<br>Descriptive and inferential statistics. SPSS. Chi-square tests.	Non-participants:<br>*N* = 1, declined participation.<br>*N* = 2, not approached due to staffing limitations.<br><br>Participants:<br>*N* = 37. Male (*n* = 20) and female (*n* = 17, 45.9%) adolescent (14 or older) paediatric oncology patients and survivors presenting for care (routine care or follow-up) at Lucile Packard Children's Hospital (LPCH) at the Stanford University Medical Center. 97 parents of patients of all ages (data not shown here).<br>Female adolescent patients of medium risk = 10/13 = 77%.<br>Type of cancers of whole adolescent patient sample:<br>• Lymphoma 43%;<br>• Leukaemia 38%;<br>• Others (neuroblastoma, rhabdomyosarcoma, osteosarcoma, spindle cell sarcoma) 19%.<br>Female adolescents: HR: 4/16; MR: 10/13; LR: 3/8.<br><br>Age at study (range) MR group (female = NR):<br>Mean 19.4, SD: 2.8 (16-25). Total group: 18.3, SD: 3 years.<br><br>Date since diagnosis (range) MR group (female = NR):<br>Mean 62.5 months, SD: 45.0 (4-129). Total group: 57 mo. (range: 3 mo-14 years).<br><br>Informed/counselled by:<br>Healthcare professionals (unclear who), including oncologists, doctors and nurses. Timing NR.
**General remarks**	**General strengths and limitations**
Exclusion criteria: patients who presented for a visit other than routine therapy or follow-up evaluation (i.e., initial diagnosis, relapse evaluation, therapy complication, palliative care).<br>15-min survey evaluated three issues: concerns about fertility-related side effects of cancer treatment, satisfaction with information provided about possible treatment effects on fertility, and the information desired for the future.<br>27 adolescents were also represented in the parent group (*n* = 34 adolescent age).	Strengths:<br>• Survey questions developed by research team and paediatric oncology physician with survey design experience, reviewed for face validity;<br>• Survey administered to two pilot samples, pilot participants provided verbal feedback about wording and clarity, items revised.<br>Limitations:<br>• Not all eligible patients included due to staffing shortage;<br>• Large proportion of participants leukaemia/lymphoma patients, limiting generalizability;<br>• Limited generalizability due to exclusion of non-English speaking families;<br>• Only information applicable for females on medium risk patients.

<table>
<tr><th colspan="2">Bias risk</th></tr>
<tr><td>
I. An explicit account of theoretical framework and/or the inclusion of a literature review which outlined a rationale for the intervention.
No: The survey was developed by a research team with experience in survey design, which was then pilot tested. However, it is not reported whether a theoretical framework or literature review was used for this.

II. Clearly stated aims and objectives.
Yes: ‘In this study, we evaluated concerns about fertility-related side effects of cancer treatment in two-samples. The satisfaction of parents and patients regarding the information provided by their oncologist regarding fertility-related side effects of cancer therapy was assessed, along with interest in learning more.’

III. A clear description of context which includes detail on factors important for interpreting results.
Yes: Information is reported on who collected data, where patients were recruited and where data was collected, when data was collected and how data was collected. Ethical approval was granted by the Stanford University Medical Center Institutional Review Board.

IV. A clear description of sample.
Yes: Age at study and date since diagnosis, gender, ethnicity, types of cancer treatment.
</td><td>
V. A clear description of methodology, including systematic data collection methods.
Yes: Information is reported on outcome measures and methodology. Data and statistical analysis is described.

VI. Analysis of the data by more than one researcher.
Not applicable to this study.

VII. The inclusion of sufficient original data to mediate between data and interpretation.
Yes: Tables provided with data on satisfaction and concerns of participants, without quotations. Discussion based on results.

Five of seven criteria met: <u>low</u> risk of bias.
</td></tr>
<tr><th colspan="2">Main outcomes</th></tr>
<tr><td colspan="2">
<u>Outcome definitions:</u>
Satisfaction defined as ‘satisfaction of parents and patients regarding the information provided by their oncologists regarding fertility-related side effects of cancer therapy’.
Regrets NR.
Concerns defined as ‘concerns of parents and patients about fertility-related side effects of cancer treatment’.
<u>Results:</u> 10 of 13 adolescents from medium risk group female.
Satisfaction (supplemental material of Oosterhuis et al.):
(Not females specific; female n = 17/37). Responses did not differ between risk categories, so categories combined. 13/37 (35.1%) adolescents satisfied with provided fertility information; thus 24/37 (64.9%) not satisfied. 14 (37.8%) received educational material, 8 (21.7%) received information on fertility preservation, 5 (13.5%) received information about where to go for information about fertility status in the future (Suppl. 2.). 17 (45.9%) want additional information by talking to a doctor who specializes in reproductive medicine, 17 (45.9%) by talking to oncology physician, 12 (32.4%) by talking to the nurse (Suppl. 3.).
Regrets: NR.
Concerns (not female specific):
Concerns of medium risk adolescents (female n = 10/13) regarding infertility (8/13), hormone production (2/13), impact on puberty (3/13) and effects on genetic material of gametes (6/13).
HR (n = 16) (not female specific n = 4/16): infertility 8, hormone production 8, impact on puberty 6, effects on genetic material of gametes 8.
LR (n = 8) (not female specific n = 3/8): infertility 3, hormone production 1, impact on puberty 1, effects on genetic material of gametes 4.
</td></tr>
</table>

Table S7: Evidence table Sandheinrich, Wondmeneh, Mohrmann, Gettinger, Henry, & Hayashi. 2018.(5)

<table>
<tr><td colspan="2">What are the experiences of female patients regarding fertility counselling during childhood cancer treatment?</td></tr>
<tr><td colspan="2">Sandheinrich T, Wondmeneh SB, Mohrmann C, Gettinger K, Henry J, Hayashi RJ. Knowledge and perceptions of infertility in female cancer survivors and their parents. Support Care Cancer. 2018;26(7):2433-9.</td></tr>
<tr><td>Study</td><td>Participants</td></tr>
<tr><td><u>Study type:</u>
Cross-sectional study.

<u>Study era:</u>
2017-2018.

<u>Country:</u>
United States of America.

<u>Data collection:</u>
Knowledge scale questionnaire, developed by fertility preservation specialists, pilot tested.
Adolescent fertility values clarification tool using Values Clarification Tool (VCT), adapted from Reproductive Concerns Scale (RCS).
Impact of event scale using Impact of Event Scale (IES).
Pediatric quality of life inventory using Pediatric Quality of Life Inventory Cancer Module (PedsQL).

<u>Data-analysis:</u>
SPSS. Descriptive statistics, patients' characteristics and outcomes scores. Pearson correlation coefficients. Two-independent t test.</td><td><u>Non-participants:</u>
NR.

<u>Participants:</u>
N = 26. Female adolescent childhood cancer survivors aged 13-18.
Seen during routine follow-up at childhood cancer late effects clinic, which sees patients >2 years after therapy.
HR = NR.
Types of cancer:
• Brain tumour 3.8%;
• Leukaemia/lymphoma 69.2%;
• Sarcoma 7.7%;
• Non-sarcomatous solid tumour 19.2%.

<u>Age at study (range):</u>
Median/Mean 16 years (13-18).

<u>Age at diagnosis (range):</u>
Median 5.25 years (0.68-15.68).
Age 0-5y: 13 (50%); 6-10: 8 (30.7%); 11-15: 4 (15.4%); 16-18: 1 (3.8%).

<u>Informed/counselled by:</u>
25/26 received FI. 9/26 participants counselled by physician (unclear if this is fertility specialist or paediatric oncologist), 11/26 by parents, 1/26 by peers and 4/26 did own research. Parents: 7/23 by physician; 11/23 own research; 5/23 peers/friends. Timing NR.</td></tr>
<tr><td>General remarks</td><td>General strengths and limitations</td></tr>
<tr><td>Aim: assess (quantitatively) knowledge and perceptions of infertility, reproductive concerns, quality of life, and emotional burden of fertility concerns in adolescent female cancer survivors and their parents.
Exclusion criteria: non-English speaking, medical records were not available for review, or documented infertility at time of study. Parents or guardians of eligible females were also eligible for participation. 23 parents participated. Patients and parents were not paired for analysis.
Mean knowledge score survivors 9.5/13 (±1.9). Did not significantly differ from mean knowledge score parents 9.96/13 ±1.7 (p = 0.394).</td><td><u>Strengths:</u>
• Use of pilot tested, validated questionnaires, scales and tools;
• Most patients counselled on potential fertility damage, meaning data are after fertility counselling;
• Female only study.
<u>Limitations:</u>
• Study data obtained from single institution, single long-term follow-up programme;
• Small, homogeneous sample, limiting generalizability;
• Most patients treated for leukaemia/lymphoma, limiting generalizability;
• No univariate/multivariate analysis, inhibiting deeper appreciation of impact.</td></tr>
</table>

<table>
<tr><th colspan="2">Bias risk</th></tr>
<tr><td>I. An explicit account of theoretical framework and/or the inclusion of a literature review which outlined a rationale for the intervention.
No: No theoretical framework or literature review were used for the intervention, but data collection was done using pilot tested or adapted scales or tools.

II. Clearly stated aims and objectives.
Yes: ‘Therefore, this study team aimed to collect quantitative data about knowledge and perception of fertility in the female paediatric cancer survivor population.’

III. A clear description of context which includes detail on factors important for interpreting results.
Yes: Information is reported on where patients were recruited, where, when and how data was collected. Ethical approval was obtained from the Institutional Review Board of Washington University in St. Louis. No information is reported on who collected data and who recruited patients.

IV. A clear description of sample.
Yes: Age at diagnosis and study, types of cancer and treatment, education and exposure to fertility information.</td>
<td>V. A clear description of methodology, including systematic data collection methods.
Yes: Information is reported on data collection, including scales and tools, and data analysis, including statistical analysis. Outcome measures are described limitedly.

VI. Analysis of the data by more than one researcher.
Not applicable to this study.

VII. The inclusion of sufficient original data to mediate between data and interpretation.
Yes: Tables with clear legends provided with findings of data from participants, without quotations. Discussion based on results.

Five of seven criteria met: <u>low</u> risk of bias.</td></tr>
<tr><th colspan="2">Main outcomes</th></tr>
<tr><td colspan="2"><u>Outcome definitions:</u>
Satisfaction not defined, but ‘I would like information about how my cancer treatment could affect my ability to have children’ and ‘I feel like I have control over my ability to have a baby’ interpreted as satisfaction.
Regrets NR.
Concerns not defined, but ‘worried’ interpreted as ‘concerns’.
<u>Results:</u>
Experience/knowledge:
Survivors score: mean 9.5/13 (SD 1.9), no significant difference from parents. 58% of survivors knows that success rate varies between fertility preservation options; 36% knows success difference between egg and embryo freezing. 50% believes chemotherapy increases risk of birth defects in offspring.
Satisfaction:
Majority (96%) wants fertility information. 85% wants a baby in the future. 69% feels frustrated about risk of infertility. 27% felt like they had control over their ability to have a baby.
Regrets: NR.
Concerns:
Over half survivors feelings of sadness/upset/depressed if they cannot have a baby (of which 6 reported devastated, mortified, depressed). 60% concerned about future child getting sick/cancer. 27% feels they have control over ability to have children. >20% concerns about having baby, possible risk of cancer recurrence.</td></tr>
</table>

Table S8: Evidence table Wright, Coad, Morgan, Stark, & Cable. 2014.(6)

What are the experiences of female patients regarding fertility counselling during childhood cancer treatment?	
Wright CI, Coad J, Morgan S, Stark D, Cable M. 'Just in case': the fertility information needs of teenagers and young adults with cancer. Eur J Cancer Care (Engl). 2014;23(2):189-98.	
**Study**	**Participants**
Study type:<br>Qualitative mixed methods study.<br><br>Study era:<br>2011-2014.<br>June 2011-February 2012.<br><br>Country:<br>United Kingdom.<br><br>Data collection:<br>Participant observation, semi-structured interviews.<br><br>Data-analysis:<br>NVivo9 software for data management and coding.	Non-participants:<br>NR.<br><br>Participants:<br>*N* = 14. Male and female young cancer survivors. Recruited from a regional evaluation in the UK, carried out in a specialist service for young people aged 13–25 years with cancer. 6 aged 12–15 at diagnosis (*n* = 2 girls), and 8 aged 18–24 at diagnosis (*n* = 3 women).<br>Female childhood cancer survivors = 5/14.<br>Female childhood cancer survivors <19 years = 4/14 = 29%.<br>Types of cancer NR.<br>HR = NR.<br><br>Age at study (range):<br>Median: NR (18-20 years). Young people were between 2 months and 4 years from finishing treatment. Female: 18, 19, 20, 20 years: median 19.5 years.<br><br>Age at diagnosis (range):<br>Median: NR (12-24 years). Female: 14, 15, 18, 18 years: median 16.5 years (1 female 20 years at diagnosis excluded).<br><br>Informed/counselled by: NR.<br>Information received on impact of cancer and treatment on fertility (3/4 females). Timing NR.
**General remarks**	**General strengths and limitations**
Most young people (*n* = NR) received mixed levels of information on fertility and counselling before treatment. 3 patterns: When diagnosed in early teens how and from whom information is received varies (*n* = NR). Young women receive incomplete information (*n* = NR). The majority (*n* = NR) is unaware of fertility status after end of treatment.<br>Paper reports challenges in ensuring AYA (13-25 years) receive comprehensive information on fertility and risk, including advice regarding fertility status after end of treatment.<br>General suggestion by Wright *et al.*: tailor-made information incorporated into practice for TYA with cancer. Including:<br>• honest and open discussions (with patients with HR infertility and unfavourable preservation, and LR of infertility);<br>• option for discussion with clinician (private, or with parents);<br>• a written summary;<br>• opportunity to reassess their fertility (standard);<br>• end of treatment clear contraception advice, pregnancy risks; reinforcement infertility might be temporary.	Strengths:<br>• Thematic content analysis, drawing out and exploring themes, allowing creation of further themes;<br>• Rich data set, illuminating unique needs of participants;<br>• Data of experiences, illustrating patterns and similarities in areas of support and improvement;<br>• Triangulation multiple perspectives, including patients, parents, carers, partners, healthcare professionals;<br>• Conceptual generalizability supported.<br>Limitations:<br>• Small sample, low generalizability;<br>• Types of cancer not reported;<br>• Differences in context between regions.

Bias risk	
**I. An explicit account of theoretical framework and/or the inclusion of a literature review which outlined a rationale for the intervention.**<br>No: No theoretical framework or literature review were used for the interview questions.<br><br>**II. Clearly stated aims and objectives.**<br>Yes: 'This paper reports on the inherent challenges that exist in ensuring young people ages between 13 and 25 years receive comprehensive information on their fertility and potential risk, along with advice on how to determine their fertility status after treatment has finished.'<br><br>**III. A clear description of context which includes detail on factors important for interpreting results.**<br>No: No information is reported on where patients were recruited, how data was collected and who collected data. Information on when data was collected is reported. thical approval was received from 'hospital paediatric and adult directorates, as well as from the university involved.'<br><br>**IV. A clear description of sample.**<br>No: Only age at diagnosis and study and gender are described. In quotations by participants cancer treatments are reported, but this information is limited to a few participants.	**V. A clear description of methodology, including systematic data collection methods.**<br>No: Limited information is reported on data collection and analysis. Outcome measures are described limitedly.<br><br>**VI. Analysis of the data by more than one researcher.**<br>No: Data analysis was performed, but it has not been specified by who this was done.<br><br>**VII. The inclusion of sufficient original data to mediate between data and interpretation.**<br>Yes: No tables have been included, but quotations of participants have been. Discussion is based on results.<br><br>Two of seven criteria met: high risk of bias.

Main outcomes
Outcome definitions:<br>Satisfaction not defined, but 'information she had been given was inadequate and needed addressing', 'additional distress' 'being given little information' and 'information she received was insufficient'.<br>Regrets NR.<br>Concerns NR.<br>Results:<br>Experiences:<br>Not female CCS specific: all but 1 (12-16 years) received information about fertility, variable satisfaction levels (*n* = NR): 1 female was informed (15y at diagnosis, 18y at study), 1 female (18y/20y) had been given limited information (oocyte cryopreservation was not available), 1 (18y/20y) received insufficient information (unclear why FP was not possible and insensitive communication and only after self-initiated conversation. 1 female (14y at diagnosis, 19y at study) was not informed and overheard peers discussing it. Physician said she was at low risk thus not informed. 3/4 were unsure of their current fertility status at time of study. Even though, 1 (15y and 18y) received excellent information, she was unsure whether she was infertile. Two other (14y/19y and 18y/20y) do not know where to go for information to find out. One (18y/20y) knows that she is infertile.<br>Conclusion: 1 was not informed and does not know fertility status, 1 was well informed, but doesn't know fertility status, two were insufficiently informed of which only 1 knows fertility status. 4/5 females raised question herself.<br>For 3 family fertility discussion worked well.<br>Satisfaction:<br>Fertility information not good, inadequate and needed addressing. Satisfied with parental support (*n* = 1). Information given insensitively caused additional distress and dissatisfaction (*n* = 1+mother).<br>Regrets: NR.<br>Concerns: NR.

Table S9: Evidence table Zarnegar, Gosiengfiao, Rademaker, Casey, & Albritton. 2018.(7)

<table>
<tr><th colspan="2">What are the experiences of female patients regarding fertility counselling during childhood cancer treatment?</th></tr>
<tr><th colspan="2">Zarnegar S, Gosiengfiao Y, Rademaker A, Casey R, Albritton KH. Recall of Fertility Discussion by Adolescent Female Cancer Patients: A Survey-Based Pilot Study. J Adolesc Young Adult Oncol. 2018;7(2):249-53.</th></tr>
<tr><th>Study</th><th>Participants</th></tr>
<tr><td><u>Study type:</u>
Observational retrospective pilot study.

<u>Study era:</u>
2013-2018.
October 2013-April 2014.

<u>Country:</u>
United States of America.

<u>Data collection:</u>
45-item online survey. Current psychological status assessed using modified six-symptom checklist for anxiety and depression (and PTSD).

<u>Data-analysis:</u>
NR.</td><td><u>Non-participants:</u>
N = 2. No data reported on non-participants.
Relapsed disease (n = 1) and declined participation (n = 1).

<u>Participants:</u>
N = 19. Female childhood cancer patients diagnosed between 13-18 years and 6 months - 3 years after diagnosis recruited from Pediatric Oncology Clinic at Lurie Children’s Hospital.
HR = NR.
Types of cancer:
Lymphoma 31.6%;
Sarcoma 31.6%;
Leukaemia 10.5%;
Ovarian tumour 10.5%;
Other 10.5%;
Brain tumour 5%.
<u>Age at study (range):</u>
NR.

<u>Age at diagnosis (range):</u>
Mean 15.6 years (13-18).

<u>Informed/counselled by:</u>
11 participants recall discussion on fertility (unclear who and if fertility specialist was involved). 8/11 initiated by doctors, nurses, psychologists, social workers or family. Timing NR.</td></tr>
<tr><th>General remarks</th><th>General strengths and limitations</th></tr>
<tr><td>Lurie Children’s Hospital has a multidisciplinary Pediatric Fertility Preservation
Program since 2008 (paediatric providers, including oncologist, oncology nurse practitioner, endocrinologist, surgeon, and urologist). A fertility specialist for oocyte cryopreservation is available at the neighbouring hospital. Ovarian tissue cryopreservation is available in-house under an investigational protocol.
Aim: validate survey do adolescent females remember fertility discussion during treatment planning, satisfied with fertility knowledge during diagnosis and treatment, identify factors influencing recall.
Recall was compared to documentation in the electronic medical record at diagnosis: 10/19 reported to not have thought about future pregnancy; 7/19 were looking forward to it, 2/9 did not want to become pregnant.</td><td><u>Strengths:</u>
First pilot study of recall of fertility discussion in adolescent females;
Findings on reasons for not undergoing fertility preservation consistent with literature for AYA patients;
Female only study.
<u>Limitations:</u>
Small sample size, limiting generalizability;
Single site with oncofertility programme, OTC options survey study bias;
Inherent recall bias;
N = 1 recalls discussion but did not answer all (satisfaction) questions, unclear why.</td></tr>
</table>

Bias risk	
**I. An explicit account of theoretical framework and/or the inclusion of a literature review which outlined a rationale for the intervention.**<br>No: No theoretical framework or literature review were used for the interview questions.<br><br>**II. Clearly stated aims and objectives.**<br>Yes: 'In this pilot study, our aim was to validate a survey to determine whether adolescent females remembered a fertility discussion during treatment a, were satisfied with their knowledge of fertility information during diagnosis and treatment, and to identify factors that may influence recall.'<br><br>**III. A clear description of context which includes detail on factors important for interpreting results.**<br>Yes: Information is reported on where patients were recruited, when, where and how data was collected. Ethical approval was granted by the Institutional Review Board of the Lurie Comprehensive Cancer Center and the Ann and Robert H. Lurie Children's Hospital of Chicago. No information is reported on who collected data.<br><br>**IV. A clear description of sample.**<br>Yes: Age at diagnosis and study, types of cancer and treatment, ethnicity, race, education and religion.	**V. A clear description of methodology, including systematic data collection methods.**<br>No: Limited information is reported on data collection and no information is reported on data analysis. Outcome measures have been described limitedly.<br><br>**VI. Analysis of the data by more than one researcher.**<br>Not applicable to this study.<br><br>**VII. The inclusion of sufficient original data to mediate between data and interpretation.**<br>Yes: Tables have been included with clear legends. Quotations were not included. Discussion based on reported results.<br><br>Four of seven criteria met: low risk of bias.

Main outcomes
Outcome definitions:<br>Satisfaction not defined, but the survey included questions on 'satisfaction with fertility knowledge during diagnosis and treatment'.<br>Regrets NR.<br>Concerns NR.<br>Results:<br>Experience:<br>Electronic records showed 10/19 fertility was discussed.<br>During initial treatment planning: 11/19 (58%) remembered infertility risk discussion (9/11 initiated by doctor, nurse, psychologist, social worker, family; 9/11 reported in chart); 8/19 not remembered (1/8 reported in chart), 9/19 (47%) fertility preservation discussion (8/9 initiated by someone else (who = NR)). 7/9 oocyte, embryo or OTC was described/offered. 4/9 hormone therapy to protect ovaries were discussed. 1/9 had OTC, 2/9 had hormone therapy, 6 declined FP. 3 thought risk having difficulty becoming pregnant in future was low; 3 said they were too young at the time, 2 did not want to delay cancer treatment, 2 did not want an experimental protocol/clinical trial, and 1 was too expensive.<br>5/19 parents made all decisions; 7/19 parents made most decisions; 7/19 made decisions with parents.<br>Satisfaction:<br>10/11 participants (90.9%) were satisfied with fertility knowledge at diagnosis and during treatment; 1/11 missing data. 5/8 participants who did not recall discussion satisfied with fertility knowledge at diagnosis and during treatment.<br>Regrets: NR.<br>Concerns: NR.

Supplementary References

1. Crawshaw MA, Glaser AW, Hale JP, Sloper P. Male and female experiences of having fertility matters raised alongside a cancer diagnosis during the teenage and young adult years. Eur J Cancer Care (Engl). 2009;18(4):381-90.
2. Jardim FA, Lopes-Junior LC, Nascimento LC, Neves ET, de Lima RAG. Fertility-Related Concerns and Uncertainties in Adolescent and Young Adult Childhood Cancer Survivors. J Adolesc Young Adult Oncol. 2021;10(1):85-91.
3. Kim J, Mersereau JE. A pilot study about female adolescent/young childhood cancer survivors' knowledge about reproductive health and their views about consultation with a fertility specialist. Palliat Support Care. 2015;13(5):1251-60.
4. Oosterhuis BE, Goodwin T, Kiernan M, Hudson MM, Dahl GV. Concerns about infertility risks among pediatric oncology patients and their parents. Pediatr Blood Cancer. 2008;50(1):85-9.
5. Sandheinrich T, Wondmeneh SB, Mohrmann C, Gettinger K, Henry J, Hayashi RJ. Knowledge and perceptions of infertility in female cancer survivors and their parents. Support Care Cancer. 2018;26(7):2433-9.
6. Wright CI, Coad J, Morgan S, Stark D, Cable M. 'Just in case': the fertility information needs of teenagers and young adults with cancer. Eur J Cancer Care (Engl). 2014;23(2):189-98.
7. Zarnegar S, Gosiengfiao Y, Rademaker A, Casey R, Albritton KH. Recall of Fertility Discussion by Adolescent Female Cancer Patients: A Survey-Based Pilot Study. J Adolesc Young Adult Oncol. 2018;7(2):249-53.
